# Amino Acid Starvation Has Opposite Effects on Mitochondrial and Cytosolic Protein Synthesis

**DOI:** 10.1371/journal.pone.0093597

**Published:** 2014-04-09

**Authors:** Mark A. Johnson, Sara Vidoni, Romina Durigon, Sarah F. Pearce, Joanna Rorbach, Jiuya He, Gloria Brea-Calvo, Michal Minczuk, Aurelio Reyes, Ian J. Holt, Antonella Spinazzola

**Affiliations:** 1 MRC-Mitochondrial Biology Unit, Wellcome Trust-MRC Building, Cambridge, United Kingdom; 2 NIMR-National Institute for Medical Research, Mill Hill, London, United Kingdom; Newcastle University, United Kingdom

## Abstract

Amino acids are essential for cell growth and proliferation for they can serve as precursors of protein synthesis, be remodelled for nucleotide and fat biosynthesis, or be burnt as fuel. Mitochondria are energy producing organelles that additionally play a central role in amino acid homeostasis. One might expect mitochondrial metabolism to be geared towards the production and preservation of amino acids when cells are deprived of an exogenous supply. On the contrary, we find that human cells respond to amino acid starvation by *upregulating* the amino acid-consuming processes of respiration, protein synthesis, and amino acid catabolism in the mitochondria. The increased utilization of these nutrients in the organelle is not driven primarily by energy demand, as it occurs when glucose is plentiful. Instead it is proposed that the changes in the mitochondrial metabolism complement the repression of cytosolic protein synthesis to restrict cell growth and proliferation when amino acids are limiting. Therefore, stimulating mitochondrial function might offer a means of inhibiting nutrient-demanding anabolism that drives cellular proliferation.

## Introduction

While mitochondria are best known for utilizing nutrients, including amino acids, for cellular energy production, they also act as a biosynthetic hub, providing precursors and substrates for anabolic pathways, such as gluconeogenesis and *de novo* synthesis of fatty acids and amino acids. In the case of amino acids, mitochondria provide oxaloacetate for the manufacture of aspartate and asparagine, and 2-oxoglutarate for glutamate, glutamine, and arginine and proline biosynthesis. Thus, mitochondria modulate amino acid homeostasis according to the particular requirements and resources of a cell. Glucose has been the most intensively studied metabolite, and it has long been known to influence oxidative metabolism and cell proliferation [1], [2]. Amino acid restriction is also well known to affect cell proliferation, yet its impact on mitochondrial function remains largely unexplored, despite the organelle’s well-established role in amino acid metabolism. Mitochondria are reported to fuse in response to amino acid starvation, presumably to protect them from autophagosomal degradation [3], [4]. However, whether amino acid deprivation affects mitochondrial function, and which mitochondrial proteins and pathways are involved in any such adaptation, has not been determined.

Mitochondria contain their own DNA (mtDNA) that encodes essential components of the oxidative phosphorylation (OXPHOS) system, and the RNA elements necessary for their translation. All of the proteins required for mitochondrial DNA replication and gene expression are encoded in nuclear DNA, synthesized on cytosolic ribosomes and imported into the mitochondria. Therefore mitochondrial responses to external stimuli, including nutrient availability, require the cooperation of nuclear and mitochondrial gene expression, yet the precise mechanisms of inter-genomic communication are only beginning to be understood [5].

Cells must respond to changes in nutrient availability in order to survive periods of starvation. The survival program includes inhibition of anabolic processes, such as protein synthesis in the cytosol, and activation of protein recycling, via autophagy and proteosomal degradation. Several kinases act as metabolic switches that respond to nutrient levels; in particular, the cellular response to amino acids is controlled by the Target of Rapamycin Complex 1, TORC1. In favorable nutrient conditions, TORC1 stimulates mRNA translation and ribosomal biogenesis, thereby promoting cytosolic protein synthesis and cell proliferation [6], [7]. Conversely, when amino acids are scarce, TORC1 activity is inhibited, resulting in down-regulation of protein synthesis in the cytosol, and quiescence [8], [9]. While many aspects of TORC1 regulation have been defined, the relationship between TORC1 and mitochondrial function is a matter of debate. Rapamycin treatment [10] or deletion of the TORC1 component Raptor [11], were associated with mitochondrial perturbations in mouse muscle; and in Jurkat T cells repression of mTORC1 was reported to decrease mitochondrial respiration [12]. On the other hand, deletion of TOR, or its target Sch9, has been shown to increase mitochondrial activity in yeasts [13], and mice lacking Raptor in adipose tissue display a higher rate of mitochondrial respiration than controls [14]. Negative regulation of mitochondrial biogenesis by TOR is also supported by work in flies, where dietary restriction elevates the level of the translation repressor d4E-BP (the eukaryotic initiation factor 4E-binding protein), a downstream target of TOR, thereby stimulating transcription in the nucleus of genes that encode mitochondrial components. [15].

Therefore, considering the importance of amino acid availability for cell proliferation, and the central role of mitochondria in amino acid metabolism and energy production, we determined the impact of these nutrients on mitochondria, mtDNA and its expression in human cultured cells. The findings lead us to propose that TORC1 activity is inversely correlated with mitochondrial protein synthesis and respiration, and that cells starved of amino acids activate catabolic processes in the mitochondria, which complement the inhibition of cytosolic protein synthesis, to halt cell proliferation.

## Results

### Amino Acid Starvation Increases Mitochondrial Respiration and Mitochondrial Membrane Potential

**Figure 1:**
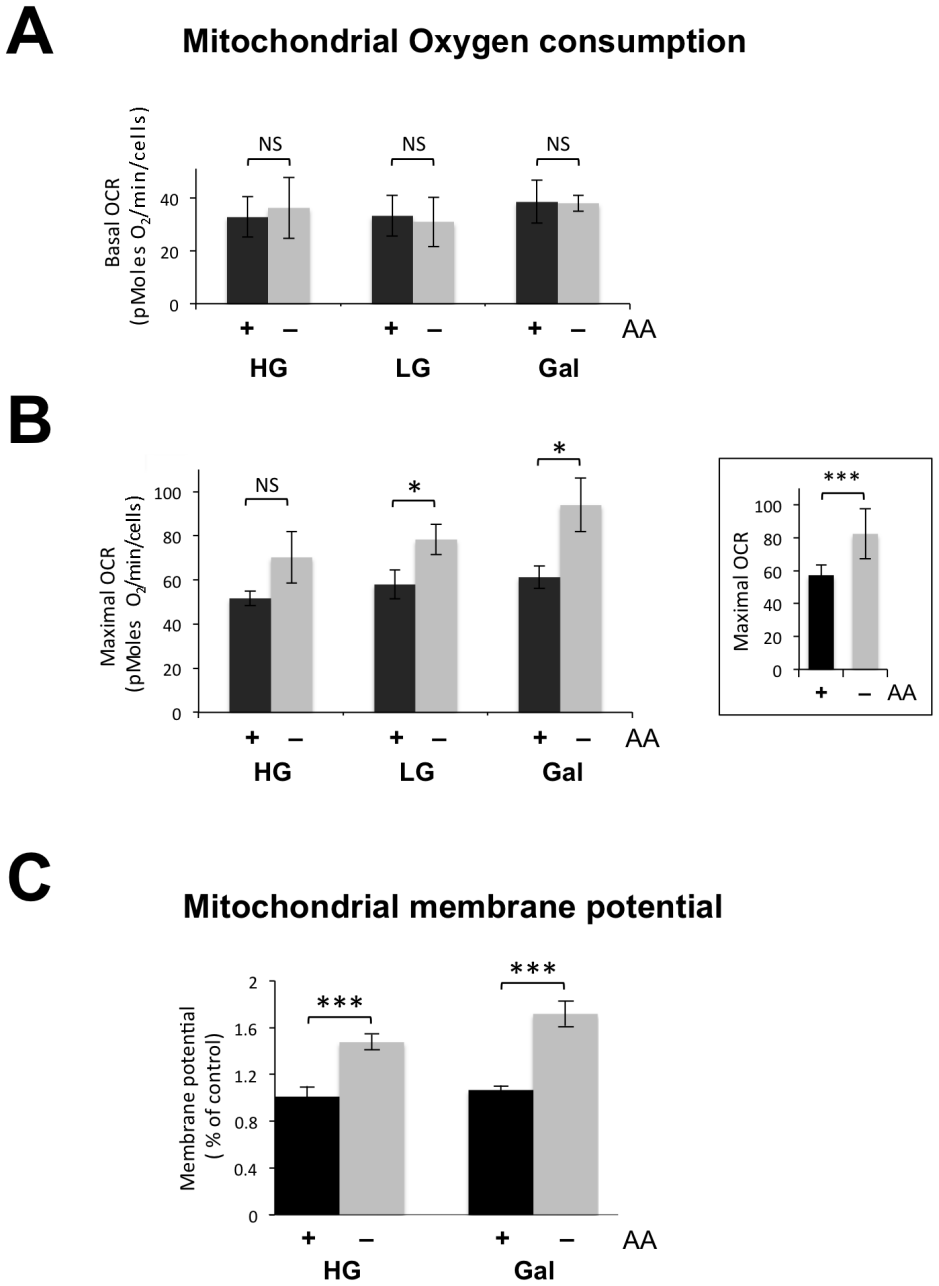
Amino acid starvation increases mitochondrial respiration and membrane potential. HEK cells were cultured in 25(HG), 5 mM glucose (LG), or 5 mM galactose (Gal), with (+) or without (−) the 15 amino acids (AA) present in the standard formulation of DMEM (Table S1). The same notation is used in subsequent figures. Mitochondrial oxygen consumption rate (OCR) was measured using a flux analyser before (basal) (**A**) and after the addition of FCCP (maximal) (**B**), having subtracted the non-mitochondrial (rotenone-insensitive) OCR. Inset shows maximal OCR of HEK cells grown in the presence or absence of amino acids combining the three sets of values for the different sugar concentrations shown in panel **B**. (**C**) Mitochondrial membrane potential was assessed by a quantitative flow cytometry analysis of TMRE fluorescence. The fluorescence values were normalized to those of HEK cells grown in HG with amino acids. The data represent the mean ± standard error of the mean (s.e.m) of 3 independent experiments, each one performed in duplicate. Statistical analysis was performed using the unpaired two-tailed Student’s *t*-test. Asterisks indicate the level of statistical significance (P<0.05 *, P<0.01 **, and P<0.001 ***); NS, not significant (p>0.05).

To determine the extent to which mitochondrial metabolism is modulated by amino acid availability in proliferating cells, and to compare and contrast it with glucose availability, we analyzed human embryonic kidney (HEK) cells grown in the presence or absence of amino acids for 72 hours, with high (25 mM) or low (5 mM) glucose, or 5 mM galactose. First, we determined oxygen consumption as a measure of mitochondrial respiratory chain capacity. Cells grown in the different conditions displayed similar basal oxygen consumption rates (Basal OCR, Fig. 1A); however, after uncoupling respiration from ATP synthesis, cells deprived of amino acids displayed increased oxygen consumption rates (Maximal OCR, Fig. 1B), irrespective of the glucose concentration. Individually the increases in the maximal OCR after amino acid withdrawal were significant (p<0.05) for low glucose and galactose, but not high glucose (Fig. 1B), and after conflating all three results the probability of the differences being due to chance decreased to p = 0.00065 (Fig. 1B, inset). Respiration drives proton translocation across the inner mitochondrial membrane, and so we next measured mitochondrial membrane potential using the Tetramethyl rhodamine ethyl ester (TMRE) fluorescent probe. Removal of amino acids from the growth medium caused the mitochondrial membrane potential to increase by ∼60% (±10%) in cells grown in HG or galactose medium (Fig. 1C). Thus, mitochondrial respiration increases in the absence of exogenous amino acids leading to elevation of the mitochondrial membrane potential.

### Amino Acid Starvation Enhances Mitochondrial Protein Synthesis

**Figure 2:**
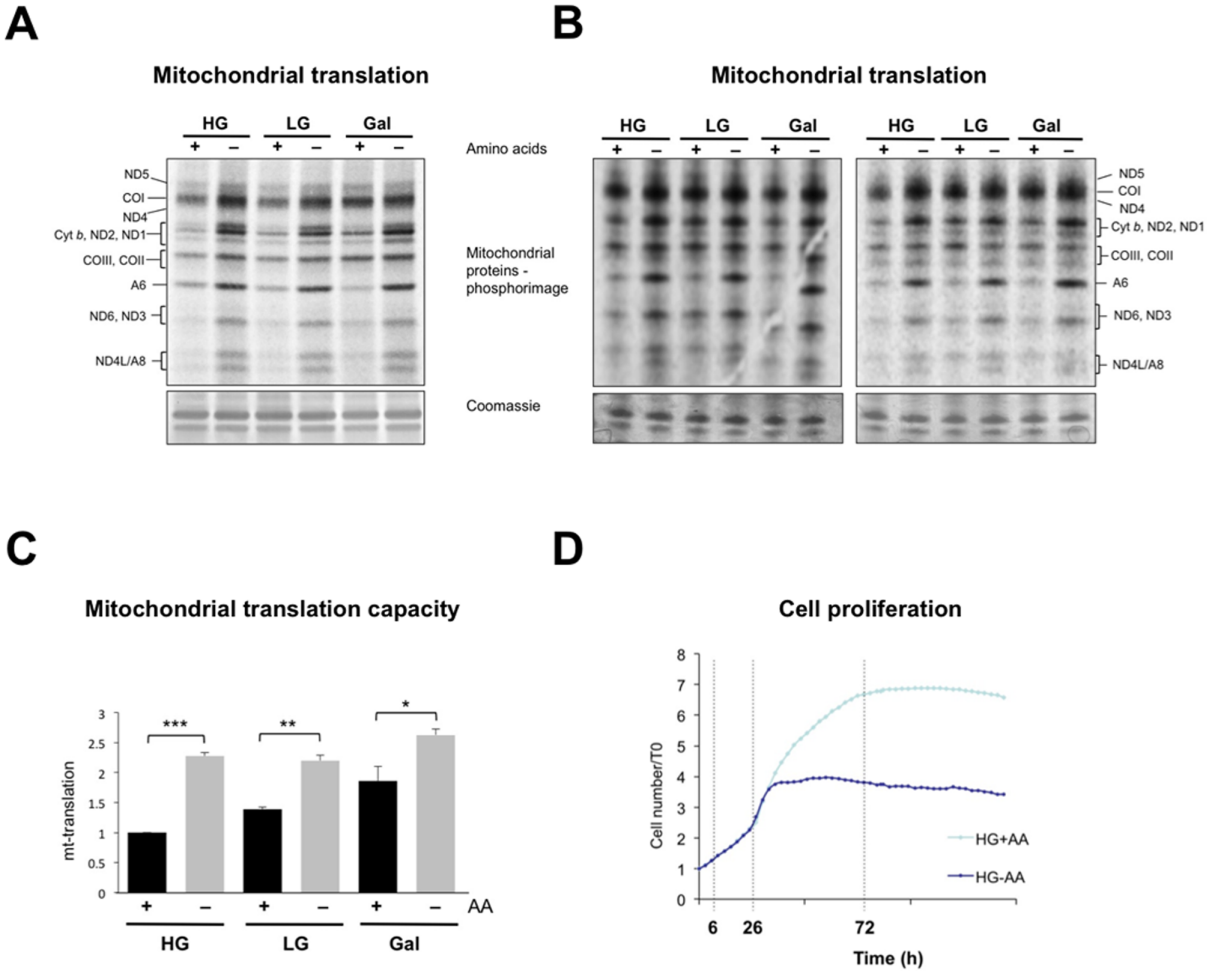
Amino acid starvation boosts mitochondrial protein synthesis. (**A**) One-hour ^35^S-methionine pulse-labeling of nascent mitochondrial polypeptides in HEK cells grown for 72 h in different media (see Figure legend 1), and fractionated by 12% SDS-PAGE. Tentative assignments of the mitochondrial polypeptides are indicated to the left of the gel: COI-III, subunits of cytochrome *c* oxidase; ND1-6 and 4L, subunits of respiratory complex I; Cyt *b* – Cytochrome *b* of respiratory complex III; A6 and A8, subunits of ATP synthase. A section of the Coomassie blue-stained gel indicates equal protein loading. (**B**) Pulse-labelings of nascent mitochondrial polypeptides and fractionation as per panel A except that the labeling medium lacked all amino acids (other than ^35^S-methionine). (**C**) The combined signal of the labeled mitochondrial proteins was quantified and normalized with respect to HG+AA. HG, n = 6 experiments; LG, n = 3 experiments; and Gal, n = 3 experiments. The error bars represent the s.e.m.; unpaired student’s *t*-test, (P<0.05 *, P<0.01 **, and P<0.001 ***). (**D**) The growth rates of cells grown in HG with or without AA were monitored and measured over the course of 7 days. Broken vertical lines at 6, 26, and 72 h indicate the times at which the mitochondrial translation capacity was measured.

The mitochondrial capacity is often enhanced via organelle biogenesis [16], which includes the synthesis of the 13 essential components of the OXPHOS system encoded by mtDNA. Mitochondrial protein synthesis might be expected to be repressed during amino acid starvation owing to the shortage of precursors, as occurs in the cytosol [6], [9]. Contrary to this expectation, the synthesis of nascent mitochondrial polypeptides was elevated after 72 hours of amino acid deprivation, whether or not amino acids were present in the labeling reaction (Figs. 2A, 2B, S1). Overall, the largest increase was the doubling of mitochondrial protein synthesis in cells cultured in high glucose in the absence of amino acids, compared to cells grown in high glucose in the presence of amino acids (Fig. 2C). Amino acid starvation inhibited cell growth after 30 hours of culture (Fig. 2D), but the increase in mitochondrial protein synthesis was already evident 6 (not shown) and 26 hours after starvation (Fig. S2). Hence, the changes in mitochondrial translation precede the growth arrest.

**Figure 3:**
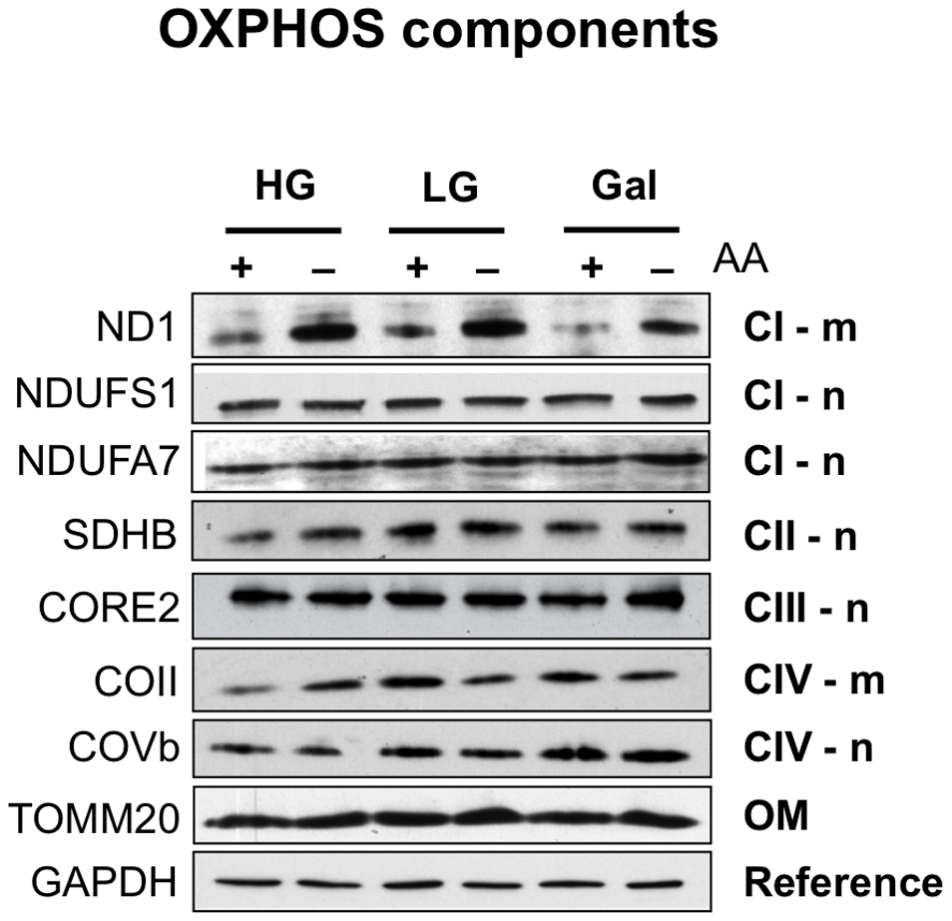
Amino acid starvation does not induce mitochondrial biogenesis. After growing HEK cells for 72: Respiratory complex I subunits, ND1, NDUSF1, and NDUFA7; complex II, succinate dehydrogenase subunit B; complex III, CORE2; complex IV, COVb and COII. TOM20, outer membrane protein of mitochondria (OM); GAPDH, reference protein. m, protein product of mtDNA; n, product of nuclear DNA.

Next, we examined whether the enhancement of mitochondrial translation led to changes in the steady-state levels of the OXPHOS components. None of the five nuclear-encoded components of the OXPHOS system screened were upregulated (Fig. 3), and the mitochondrial mass was unaltered, as determined by the abundance of the outer mitochondrial membrane protein, TOM20 (Fig. 3). With respect to mitochondrially encoded proteins, amino acid deprivation induced a marked increase in the level of a subunit of respiratory complex I (ND1), but no increase in cytochrome c oxidase subunit II (Fig. 3). These findings suggest that the enhanced respiration induced by amino acid starvation is not the result of wholesale mitochondrial biogenesis but depends on a subset of OXPHOS components.

### Amino Acid Deprivation Increases the Levels of Many but not All Mitochondrial Transcripts

**Figure 4:**
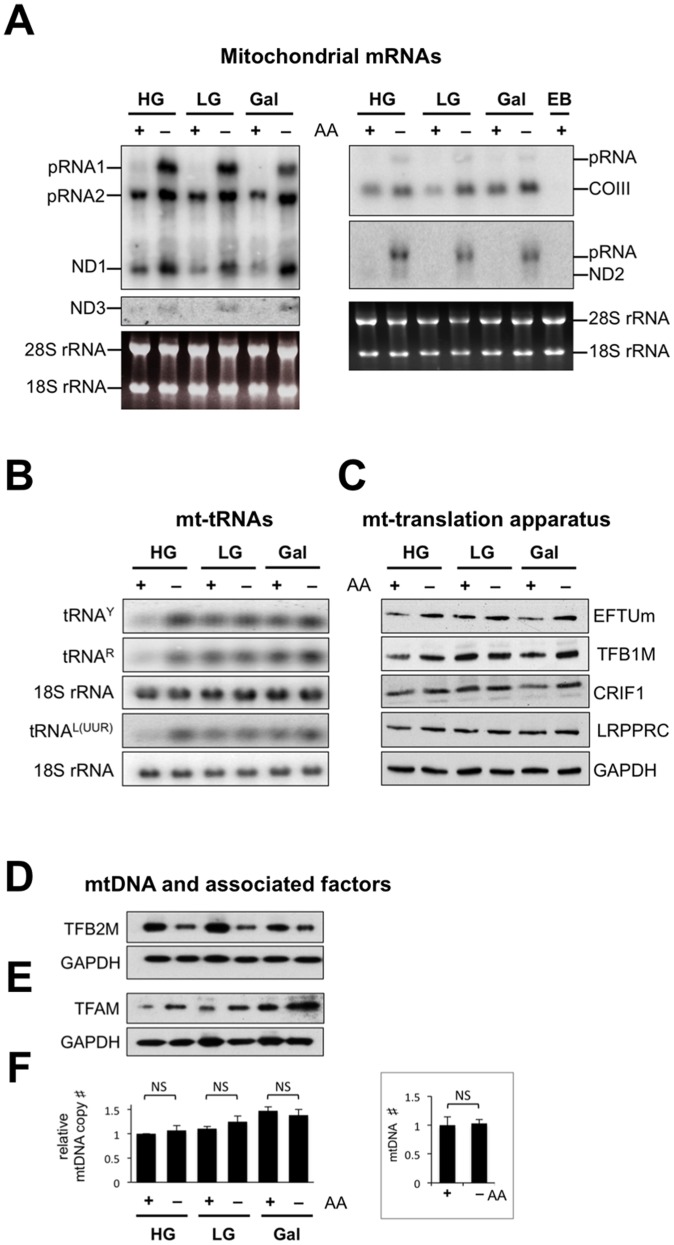
Amino acid deprivation increases the levels of several mitochondrial RNAs, their protein products, and TFAM and EFTu. DNA, RNA, and protein were harvested from HEK cells grown for 72(**A**) and (**B**) Northern hybridization with probes corresponding to mRNAs and tRNAs, respectively. COIII, cytochrome oxidase III mRNA; ND1-3, RNAs encoding subunits of complex I; pRNA, precursor RNA; EB, ethidium bromide. 18S and 28S rRNA are the RNA elements of cytosolic ribosomes. R – arginine, Y – tryptophan, L^(UUR)^ – Leucine. (**C**) Immunoblots of selected mitochondrial translation machinery factors: EFTUm – mitochondrial translation elongation factor; TFB1M, modifies the RNA of the small subunit of the mitochondrial ribosome; CRIF1, a contributor to normal mitochondrial translation; LRPPRC, a mitochondrial mRNA-interacting protein. (**D**) and (**E**) Immunoblots of TFAM and TFB2M, respectively. (**F**) Q-PCR estimation of mtDNA copy number; n = 6; error bars represent the s.e.m. There was no significant change in mtDNA copy number as a result of amino acid withdrawal based on the unpaired student’s *t*-test, NS not significant (p>0.05), for any sugar concentration, or all (inset). Using the same test there was a significant increase in the mtDNA copy number when glucose was replaced by galactose (Figure S6).

Mitochondrial DNA yields three classes of RNAs, messenger, ribosomal, and transfer RNAs, increases in any of which might enhance mitochondrial protein synthesis. Northern analysis of six mitochondrial mRNAs (ND1, ND2, ND3, COII, COIII, and A6/A8, the last encoding two subunits of ATP synthase) revealed increases in the precursors and mature transcripts, with the exception of COII, as a result of amino acid, but not glucose, limitation (Figs. 4A, and S3A). Amino acid withdrawal elicited no such increase in ribosomal RNAs, or in three protein components of mitochondrial ribosomes (Fig. S3B), whereas the levels of transfer RNAs increased when the availability of amino acids or glucose was restricted (Figs. 4B, and S3C). Among selected factors required for mitochondrial protein synthesis, only the mitochondrial translation elongation factor EFTu strictly correlated with amino acid deprivation (Fig. 4C). Because EFTu facilitates the insertion of aminoacyl-tRNAs into the A-site of the ribosome [17], its upregulation could allow for more efficient delivery of charged tRNAs to mitochondrial ribosomes, potentially making it a key part of the mechanism by which translation is stimulated in the absence of exogenous amino acids.

The increases in mature and precursor RNAs indicate that mitochondrial RNA metabolism responds to amino acid availability. However, the differences in the level of mature transcripts, which included increases in ND1-3, COIII and A6/A8, a decrease in 12S rRNA and unaltered COXII and 16S rRNA, do not fit a simple model in which amino acid starvation stimulates mitochondrial transcription. Moreover, a key mitochondrial transcription factor (TFB2M) [18], [19] was repressed by amino acid starvation, and the mitochondrial RNA polymerase was unchanged (Figs. 4D, and S3D). Thus, the observed increases in many mature mitochondrial transcripts, owing to amino acid deprivation, are probably due to reduced RNA turnover, rather than increased RNA synthesis. Mitochondrial biogenesis induces increases in mtDNA copy number that are accompanied by increases in the mtDNA packaging protein TFAM (mitochondrial transcription factor A) [20]. Although the amount of TFAM increased in response to amino acid withdrawal (Figs. 4E, and S3D), there was no change in mtDNA number (Fig. 4F), whereas others have noted a strict correlation between the two [21].

### Amino Acid Starvation Increases the Capacity of Mitochondria to Catabolize Amino Acids

**Figure 5:**
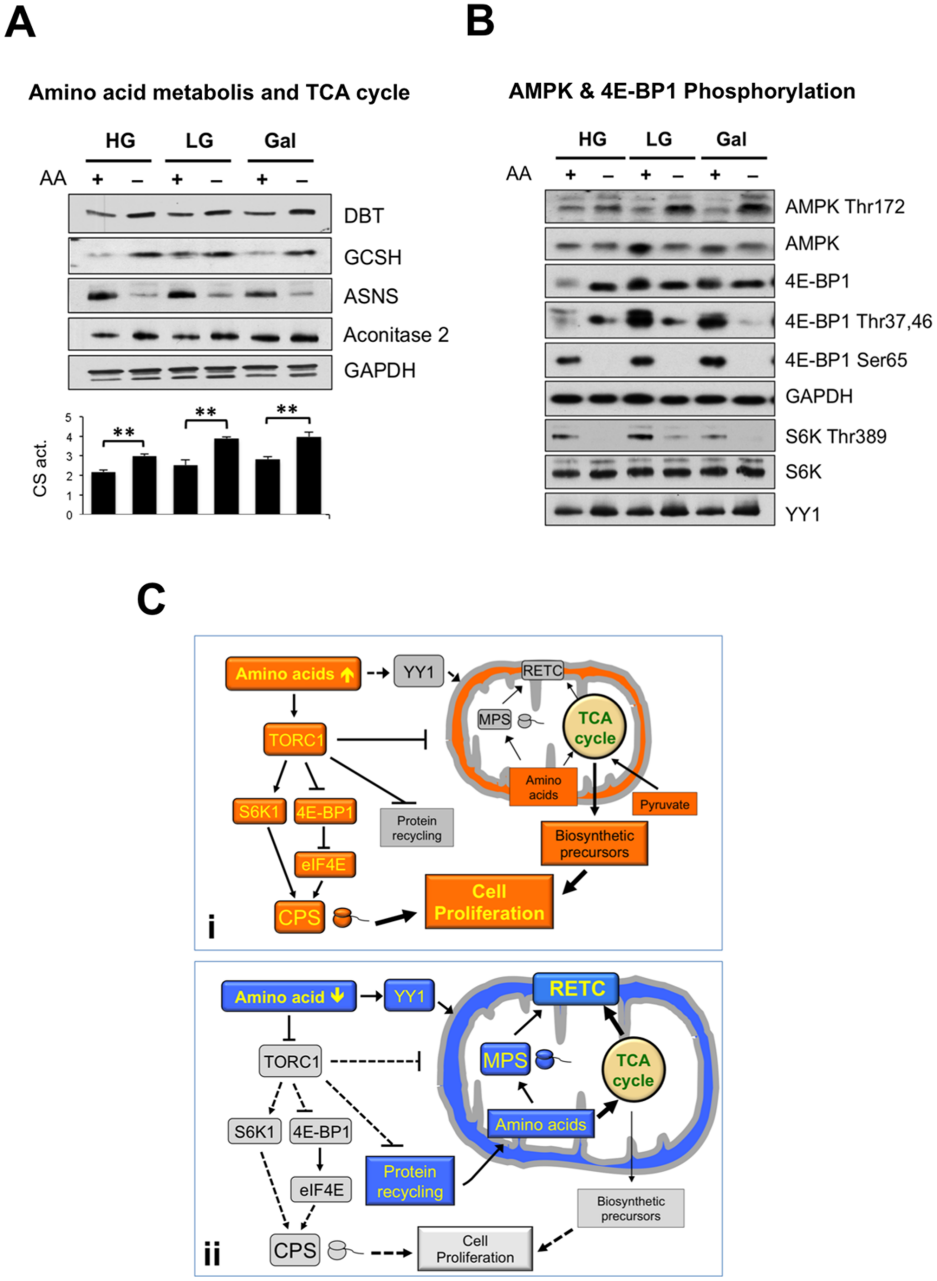
HEK cells starved of amino acids have elevated levels of amino acid-catabolizing and TCA cycle enzymes and YY1, while displaying the signature of TORC1 inhibition. HEK cell extracts were immunoblotted for (**A**) proteins involved in mitochondrial amino acid metabolism (DBT, GCSH, ASNS, see text for details) and aconitase 2. The chart shows the citrate synthase enzyme activity in the cell lysates (nmol citrate/s/mg protein). Error bars represent the s.e.m.; n = 3 (pairs); two asterisks, p<0.01. (**B**) The abundance of sensors and effectors linked to nutrient signaling in the different growth regimes was determined by immunoblotting for AMPK, 4E-BP1, S6K, and YY1, with GAPDH as reference. The reference corresponding to the blots of S6K and YY1 is not shown. The numbers following the amino acids indicate the key phosphorylated residues for which the antibody is specific. (**C**) schemes illustrating the influence of mitochondria on cellular anabolism (i) and catabolism (ii) according to amino acid availability. i) to proliferate cells require amino acids from the breakdown of food, in these conditions TORC1 is active; it promotes cytosolic protein synthesis (CPS) and inhibits protein recycling, mitochondria make relatively little contribution to energy production (which is more reliant on glycolysis – not shown). Conversely, amino acid deprivation (ii) inhibits TORC1, which leads to the repression of cytosolic protein synthesis (CPS) and the upregulation of mitochondrial protein synthesis (MPS) and the respiratory electron transport chain (RETC). YY1 also contributes to increased mitochondrial respiration, either independently as illustrated, or possibly via derepression owing to TORC1 inhibition.

Because mitochondria play a major role in amino acid homeostasis, we next examined this aspect of mitochondrial function. The withdrawal of exogenous amino acids increased the capacity of mitochondria to catabolize endogenous amino acids, as demonstrated by the increased levels of glycine cleavage system protein H (GCSH), an enzyme involved in glycine degradation, and DBT, the E2 subunit of branched chain keto-acid dehydrogenase that breaks down essential branched chain amino acids (Fig. 5A). Conversely, the expression of asparagine synthetase (ASNS), a mitochondrial enzyme devoted to the synthesis of asparagine, was markedly repressed (Fig. 5A). These data suggest somewhat paradoxically that amino acid starvation leads to increased amino acid breakdown in mitochondria. The process necessitates further oxidation via the tricarboxylic acid (TCA) cycle, which was also upregulated in response to amino acid withdrawal, as assessed by citrate synthase activity and aconitase 2 expression (Fig. 5A).

### Protein Synthesis in the Cytosol and in the Mitochondria are Reciprocally Co-regulated

To investigate the cell signaling pathways that might be involved in the mitochondrial response to amino acid starvation, we studied two well-known energy and nutrient sensors AMP-activated protein kinase (AMPK) and TORC1 [6], [22], together with two recognized inducers of mitochondrial biogenesis, the transcription co-activator PGC1α [23], [24] and the transcription factor YY1 [23], [25]. Although there was an increase in active AMPK whenever amino acids were withdrawn from the growth medium, the extent of the increase was dependent on sugar availability (Fig. 5B). Thus, AMPK activation did not parallel the observed increase in mitochondrial energy producing capacity. Mitochondrial respiration and protein synthesis were also not strictly correlated with PGC1α expression, which was increased when amino acids or glucose were limited (see Fig. S4). In contrast to AMPK and PGC1α, YY1 expression responded to amino acid, and not to glucose, availability (Fig. 5B).

When mitochondrial function was enhanced by amino acid deprivation TORC1 was inactive, as evidenced by the hypophosphorylated states of its downstream substrates, p70 S6 kinase (S6K) [26] and 4E-BP1 [27] (Fig. 5B). Hypophosphorylated S6K and 4E-BP1 are indicative of the downregulation of cytosolic protein synthesis. Thus, amino acid starvation in human cells stimulates mitochondrial protein synthesis while repressing translation in the cytosol, suggesting that protein synthesis in the two compartments is reciprocally regulated (Fig. 5C). If this is true, then repressing key factors contributing to mitochondrial protein synthesis might upregulate cytosolic translation. To test this concept, we compared the abundance of the 12, 16 and 18S RNA elements of mitochondrial and cytosolic ribosomes after repressing the expression of two proteins that contribute to mitochondrial translation. Gene silencing of METTL17 and CHCHD1 in human osteosarcoma cells (which inhibits mitochondrial translation; [28] and He and Holt, unpublished findings) is accompanied by an increase in the abundance of the 18S rRNA component of cytosolic ribosomes (see Fig. S5 A and B), thereby bolstering the idea that mitochondrial and cytosolic protein synthesis are subject to oppositely acting co-regulators.

## Discussion

Proliferation requires energy, nutrients and a swath of biosynthetic activities to duplicate the contents of the cell. When cells switch from a non-dividing to a dividing state they increase their reliance on glycolysis, a phenomenon known as the Warburg effect [29]. Although the Warburg effect occurs early in carcinogenesis, and so may predispose the cell to malignant transformation [30], it was widely overlooked by cancer biologists for over half a century [31]. The heavy dependence of cancer cells on glycolysis for energy production, when respiration is far more efficient at producing ATP, appeared paradoxical. However, much later it was appreciated that glycolysis provides important biosynthetic (anabolic) precursors for cell proliferation. Here we have revealed a situation that can be interpreted as the opposite of the Warburg effect; cells starved of amino acids increase the capacity of the mitochondria to respire and to catabolize amino acids. There is evidently no shortage of amino acids in the mitochondria in these conditions as they synthesize proteins at a higher rate than normal (Figs. 2A-2C and S1 and S2). To achieve an increase in mitochondrial protein synthesis when exogenous amino acids are lacking, the cell must channel the products of protein turnover to the mitochondria (Fig. 5C). This is compatible with the amino acids being required for energy production, and the upregulation of amino acid catabolizing enzymes in the mitochondria of cells starved of amino acids is consistent with this idea. However, these changes occur in conditions where an alternative energy source, glucose, is present in abundance. Therefore we conclude that the consumption of amino acids in mitochondria when cells are deprived of an exogenous supply is not driven by a need for energy. Instead we propose it serves primarily to restrict anabolism and thereby to halt cell proliferation. Thus, enhancing catabolism via increased mitochondrial respiration might be as important as inhibiting cytosolic protein synthesis in restricting uncontrolled cellular proliferation.

Amino acid deprivation inhibits TORC1 activity and cytosolic protein synthesis, whereas it increases mitochondrial translation and respiration in human embryonic kidney cells. This represents a new finding in human cells and supports the studies that in lower organisms suggested active TOR represses mitochondrial function [13]–[15]. In contrast to AMPK and PGC1α, the enhanced mitochondrial respiration and membrane potential associated with TORC1 inhibition are not the result of an overall increase in mitochondrial biogenesis, rather it is more narrowly focused on mitochondrial translation. Thus, it is not necessary to replace old mitochondria with new [32]; instead, the findings of this report suggest that the substitution of a subset of organelle components is sufficient to increase the energy transducing capacity of the mitochondria. Inversely, there can be selective turnover of particular components of mitochondria, such as complex I, leading to energy insufficiency [33].

Although the steady-state level of the peroxisomal protein PMP70 (Fig. S1C) was markedly decreased after seventy-two hours of amino acid deprivation, this growth regime did not lead to an appreciable decrease in GAPDH or total protein content per cell (Fig. S1). Hence, the increases in mitochondrial amino acid consumption reported here may be fueled more by extra-cellular, than by intracellular, proteins. The viable cells deprived of amino acids could source protein either via phagocytosis of cellular debris or pinocytosis-mediated internalization of serum proteins.

Citrate synthase activity is widely used as a measure of mitochondrial biomass. The finding that it can increase (Fig. 5A) with no concomitant increment in the steady-state level of a range of OXPHOS components or TOM20 (Fig. 3) indicates that citrate synthase is not always a reliable indicator of mitochondrial mass.

The data on TFAM have important ramifications for fundamental aspects of mtDNA maintenance. Previously, it has been shown that the expression of TFAM correlates strictly with mtDNA copy number [21]. However, in the current study the upregulation of TFAM in response to amino acid starvation elicited no appreciable effect on mtDNA levels (Fig. 4F). The discordance between the levels of TFAM and mtDNA observed here is mirrored in yeasts, where levels of the TFAM homologue Abf2 increased 27-fold in response to the TOR inhibitor rapamycin [34], and the deletion of TOR, while stimulating mitochondrial respiration, had no effect on the mtDNA copy number [13]. Hence, both TFAM and Abf2 are implicated in the stimulation of mitochondrial energy production independent of mtDNA maintenance.

OXPHOS deficiency induces an amino acid starvation-like response in a mouse model of mitochondrial disease [35], suggesting that respiratory-deficient cells remodel amino acid metabolism to limit the loss of mitochondrial function. Thus, the inverse correlation between TORC1 activity and mitochondrial translation and TCA cycle activity has potentially important implications for mitochondrial diseases; i.e., inhibiting TORC1 might provide a novel means of enhancing mitochondrial function in pathological states. However, treatment of mouse myotubes for 14 hours with the TOR inhibitor rapamycin suppressed a number of nuclear genes encoding mitochondrial components, and marginally reduced mitochondrial respiration, by provoking the dissociation of PGC1α from the YY1-TORC1 complex [11]. Then again, extending the rapamycin treatment to 96 hours reversed the effect on PGC1α, and increased the expression of YY1, and TFAM and an OXPHOS component, compared to controls [36]. Thus, the longer rapamycin treatment produced effects more like those reported here for amino acid starvation; nevertheless, there was no increase in mitochondrial respiration [36]. Hence, YY1 and TFAM elevation appear to be necessary but not sufficient to boost mitochondrial function, which may additionally require increased PGC1α levels. Therefore, rapamycin alone may not benefit patients with mitochondrial diseases.

Finally, the reciprocal relationship between cytosolic and mitochondrial translation identified herein helps to explain the many studies that have linked dietary restriction, mitochondrial function, and longevity [2], [13], [15], [20], [37], [38]. Viz. upregulation of mitochondrial function induces a starvation-like response, which in conjunction with repressed cytosolic protein synthesis, restricts cell growth. This may ‘slow the clock’ with respect to aging, as many cellular processes are suspended or downregulated.

## Materials and Methods

### Cell Culture

Human embryonic kidney cells (HEK293T, Invitrogen) and 143B osteosarcoma (HOS) (ATCC, CRL-8303) cells were maintained in Dulbecco’s Modified Eagle’s Medium (DMEM) containing 25 mM glucose, a full complement of amino acids and 10% fetal bovine serum. For the experiments cells were grown for 6, 26 or 72 h in DMEM containing 10% dialyzed serum and high (25 mM) or low (5 mM) glucose, or 5 mM galactose, with or without amino acids (Table S1). Incubations started 24 h after passaging the cells and they were harvested at 80% of confluency.

### Q-PCR Estimation of mtDNA Copy Number and PGC1α Transcript in HEK293T Cells

Q-PCR was performed on 25 ng lots of total cellular DNA, using portions of the COII and cytochrome *b* genes for mtDNA and APP1 for nuclear DNA. Primers with the following sequences were employed: COXII, forward 5′-CGTCTGAACTATCCTGCCCG-3′, reverse 5′-TGGTAAGGGAGGGATCGTT G-3′, probe 5′-CGCCCTCCCATCCCTACGCATC-3′; Cytb forward 5′-GCCTGCCTGATCCTCCAAAT-3′, reverse 5′-AAGGTAGCGGATGATTCA GCC-3′, probe 5′-CACCAGACGCCTCAACCGCCTT-3′. Probes contained a 5′ FAM fluorophore and a 3′ TAMRA quencher (Sigma Genosys). For the nuclear reference gene a validated (20 x) APP TaqMan Copy Number Assay master mix was used (Applied Biosystems ID Hs00339475_cn) containing primers and probe. A 2× TaqMan Gene Expression master mix (Applied Biosystems) was used in all reactions. Cycle conditions were the default setting on the ABI sequence detection system 7700. PGC1α mRNA abundance was estimated relative to the mRNA for GAPDH. Total RNA was extracted from cells with Trizol reagent (Ambion) and PureLink RNA Mini Kit (Ambion). cDNA was generated with an Ominiscript reverse transcription kit (Qiagen) according to the manufacturer’s instructions. PGC1α mRNA was performed using validated TaqMan Gene Expression Assay mix ID Hs01016719_m1 (Applied Biosystems). GAPDH levels were quantified as described previously [39]. All experimental data were analyzed using the ΔΔCt method to generate relative values for experimental versus control. Control samples were HEK cells grown in standard DMEM (25 mM glucose and a full complement of amino acids).

### Mitochondrial Translation

The labeling of mitochondrial translation products with ^35^S-methionine was performed as described previously [40] in DMEM containing 25 mM glucose, with or without amino acids (Table S1). Briefly, cells were incubated with 100 μg/mL emetine for 20 min to inhibit cytosolic protein synthesis, before adding ^35^S-methionine for 1 h, after which cells were lysed and equal amounts of protein separated by 4–12 or 12% SDS-PAGE. Gels were dried and exposed to phosphor plates, and the signals quantified with a TyphoonTM Phosphorimager (GE Healthcare).

### Immunoblotting

Cells were counted and lyzed on ice in TG buffer (Tris.HCl 20 mM pH 7.5, NaCl 500 mM, EDTA 2 mM, Triton-X-100 1%, Glycerol 10%, protease inhibitors (Roche) and phosphatase inhibitors (Na_3_VO_4_ 1 mM, NaF 50 mM, β-Glycerophosphate 10 mM). Lysate was centrifuged at 13000×g and supernatant transferred to fresh pre-chilled 1.5 mL tubes. Protein concentration was determined by BCA assay (Pierce). Equal amounts of protein or 2.5×10^4^ cells were separated on 4–12% or 12% Bis-Tris NuPAGE gels (Life Technologies). Protein was transferred to PVDF membrane then blocked in 5% milk/PBST for 1 h. Immunoprotein detection utilized antibodies to mouse anti-Aconitase 2 (1∶1000, Abcam), rabbit anti-AMPK (1∶1000, Cell Signaling), rabbit anti-phospho-AMPK Thr 172 (1∶500, Cell Signaling), mouse anti-ASNS (1∶1000, Abcam), mouse anti-COII (1∶2000 Mitoscience), rabbit anti-COVb (1∶2000) was a kind gift of J. Walker, mouse core2 (1∶1000, Invitrogen), rabbit anti-CRIF1 (1∶2000) was raised against recombinant protein produced in-house), mouse anti-DBT (1∶1000 Abnova), rabbit anti-cytochrome oxidase II (1∶1000, Mitoscience), mouse anti-GAPDH (1∶1000 GeneTex), rabbit anti-GCSH (1∶1000 Proteintech), rabbit anti-mtRNAP (1∶1000, Abcam), rabbit anti-LRPPRC (1∶1000 Santa Cruz), rabbit anti-MRPL12 (1∶5000, Abcam), rabbit anti-MRPS18 (1∶1000, Proteintech), rabbit anti-MRPS29 (1∶1000, Abcam), rabbit anti-NDUFS1 (1∶4000), chicken anti-NDFA11 B14.7 (1∶2000) and chicken anti-ND1 (1∶4000) were kind gifts of J.Walker, mouse anti-PMP70 (1∶1000, Sigma), rabbit anti-p70 S6K (1∶1000, Cell Signaling Technology), rabbit anti-phospho-p70 S6K Thr 389 (1∶1000, Cell Signaling Technology), mouse anti-SDHB (1∶250 Abcam), rabbit anti-TFAM (1∶20,000) was kind gift of R. Wiesner, goat anti-TFB1M (1∶1000, Thermo Scientific), goat anti-TFB2M (1∶1000, Abcam), rabbit anti-TOM20 (1∶20,000, Santa Cruz), mouse anti-EFTUm (1∶1000, Abnova), rabbit anti-YY1 (1∶2000, Bethyl Labs), rabbit anti-4E-BP1 (1∶1000, Cell Signaling Technology), rabbit anti-phospho-4E-BP1 Thr37,46 (1∶500, Cell Signaling Technology), rabbit anti-phospho-4E-BP1 Ser65 (1∶500, Cell Signaling Technology). Secondary antibodies were anti-chicken HRP (1∶1000 Promega), anti-mouse HRP and anti-rabbit HRP (1∶3000 Promega) or anti-goat HRP (1∶1000 Santa Cruz).

### RNA Extraction and Northern Blotting

Total RNA from HEK293T cells was extracted using Trizol (Invitrogen) via chloroform extraction and isopropanol precipitation according to manufacturer’s specifications. 5–8 μg of total RNA was resolved on agarose gels (1.2% agarose, 1.17% (0.39 M) formaldehyde, 1x MOPS) at 80V for 3 h 30 min, in 1X MOPS buffer supplemented with 0.78% (0.26 M) formaldehyde and 10 μg/ml ethidium bromide. (1X MOPS: 20 mM MOPS, 5 mM Na acetate, 1 mM EDTA). Resulting gels were imaged under UV. RNA was transferred onto MagnaProbe nylon membrane (GE) in 5X SSC, 10 mM NaOH and RNA was UV-crosslinked to the membrane. Membranes were either probed with radioactively labeled PCR fragments or with T7 promoter derived riboprobes. PCR products were labeled with ^32^P-dCTP (Hartmann Analytic) using DNA Polymerase I Klenow Fragment (New England Biolabs). Riboprobes were labeled with ^32^P-UTP (Hartmann Analytic) using the Maxiscript T7 kit (Ambion).

Forward and reverse primers for probes were as follows 5′-3′: ND1, CATGGCC AACCTCCTACTCCTCATT and GGCAGGAGTAATCAGAGGTGTTCTTG; A6/COII, TATTCCTAGAACCAGGCGACCTGC and TTTCGTTCATTTTGGT TCTCAGGGTTG; COX3, TGACCCACCAATCACATGCCTATCATATAG and GACCCTCATCAATAGATGGAGACATACAG; ND2, CTGCCATCAAGTATT TCCTCACGC and TCAGGTGCGAGATAGTAGTAGGGTC; ND3, GTATGT CTCCATCTATTGATGAGGGTCTTAC and TGTAGTCACTCATAGGCCAGA CTTAG; ATP8/6 CACCCAACAATGACTAATCAAACTAACCTC and TATGAGGAGCGTTATGGAGTGGAAG; 28S rRNA GCCTAGCAGCCGA CTTAGAACTGG and GGGGCCTCCCACTTATTCTACACC; 18S rRNA GTTGGTGGAGCGATTTGTCT and GGCCTCACTAAACCATCCAA. Riboprobes for mt-tRNAs were: Leu^UUR^ TAATACGACTCACTATAGGGA GACTGTTAAGAAGAGGAATTGAACCTCTG and GTTAAGATGGCAGAGC CCGG; Tyr TAATACGACTCACTATAGGGAGACTGGTAAAAAGAGGCC TAACCC and GGTAAAATGGCTGAGTGAAGC; Arg TAATACGACTCACTA TAGGGAGACTTGGTAAATATGATTATCATAATTTAATG and TGGTAT ATAGTTTAAACAAAACGAA.

### Mitochondrial Respiration, Membrane Potential and Citrate Synthase Activity

Mitochondrial respiration was assayed in triplicate in HEK293T cells on 24 well microplates, using a XF24 Extracellular Flux Analyzer (Seahorse Bioscience), 24 h after seeding. The wells containing cells were sequentially injected with 20 mM 2-deoxyglucose (2-DG) to inhibit glycolysis, 100 nM oligomycin to inhibit ATP synthase, 500–1000 nM carbonylcyanide-4-trifluorometho-xyphenylhydrazone (FCCP) to uncouple the respiratory chain and 200 nM rotenone to inhibit complex I. For membrane potential measurements, 5×10^5^ cells were incubated with 100 nM tetramethylrhodamine ethyl ester (TMRE) (Invitrogen) in PBS for 1 h at 37°C, trypsinized, resuspended in PBS, placed on ice in the dark and analyzed without delay by flow cytometery (BD LSRII). Debris and apoptotic cells were excluded from the analyses using forward and side scatter gating. Acquired data were analyzed by FlowJo software (Tree Star, Inc.). Three independent experiments were performed in triplicate, each based on 50,000 events. The results for cells deprived of amino acids were normalized to the values for cells cultured in medium containing amino acids. In control experiments, FCCP-treatment produced a collapse in the membrane potential based on TMRE measurements and cells lacking a respiratory chain (rho zero cells) had markedly lower membrane potential than controls (data not shown). Citrate synthase activity was assayed as previously described [41].

### Cell Proliferation

Cell proliferation rate was determined using an IncuCyte live-cell imaging system (Essen Instruments). Twenty four hours after seeding 1.5×10^5^ cells in triplicate, the medium was changed and phase-contrast images were acquired at 3 h intervals over a period of 5–7 days, and processed automatically.

### Statistical Analysis

Data were expressed as mean, standard error of the mean (SEM) or standard deviation (SD). Probability was determined using a two-tailed, unpaired Student’s *t*-test.

## Supporting Information

Figure S1 **Amino acid deprivation induces an increase in mitochondrial translation per cell.** (**A**) After labeling of newly synthesized mitochondrial proteins (as per Fig. 2 and methods), HEK cells were harvested and the cell number counted. Lysate from 2.5×10^4^ cells were separated by 12% SDS-PAGE (**A** and **B**). The protein in the gels was stained with Coomassie blue post-electrophoresis and after drying the labeled mitochondrial proteins were detected using a Phosphorimager. The total amount of protein was similar for 2.5×10^4^ cells irrespective of the presence or absence of amino acids in the growth medium, based on Coomassie blue staining. However, some individual proteins changed markedly in response to amino acid deprivation, the steady state level of ND1 increased (Fig. 3), whereas a peroxisomal protein (PMP70) decreased (**C**), in line with a previous study [3], [42].
(TIF)

Figure S2 **Enhanced mitochondrial translation in response to amino acid starvation.** (**A**) An example of the mitochondrial translation products of cells grown+or – amino acids (AA), in high (HG) or low glucose (LG), or galactose (Gal) for 26 h, fractionated by 4–12% SDS-PAGE. Below each autoradiogram is a section of the corresponding Coomassie blue-stained gel to show equal protein loading. (**B**) The signal profile from each lane in panel B was measured by phosphorimaging and the traces for+(black line) and - (gray line) amino acids aligned to give a direct comparison of the relative abundance of the mitochondrial polypeptides; HG, high glucose, LG, low glucose, Gal, galactose. Tentative assignment of the polypeptides is as per Fig. 2A.
(TIFF)

Figure S3 **Effects of amino acid deprivation on mitochondrial RNAs and proteins involved in mitochondrial transcription.** RNA and proteins were extracted from HEK cells. The RNA was fractionated by 1.2% agarose gel electrophoresis, transferred to nylon membranes, and hybridized with probes corresponding to mRNAs (**A**), rRNAs (**B**), or tRNAs (**C**). The proteins were immunoblotted using antibodies against (**B**) three mitochondrial ribosomal components (MRPs) or (**D**) the core transcription apparatus. (**A**) COII – cytochrome c oxidase subunit II mRNA, A6/A8– the single mature mRNA that encodes two subunits of ATP synthase. (**B**) 16S and 12S rRNAs are the RNA elements of mitochondrial ribosomes. (**C**) To gain an overall impression of the level of tRNAs in cells grown with or without amino acids, the portion of the membrane where tRNAs reside was hybridized to two labeled probes that together span the entire mitochondrial genome, as previously described [43]. In (**A**) and (**C**), the images for+and – amino acids derive from different portions of the same gel; additional samples (not shown) occupied the intervening lanes. **(D)** Immunoblotting for HEK cellular proteins indicated that amino acid deprivation had no effect on the steady state level of the mitochondrial RNA polymerase, in contrast to TFAM and TFB2M. GAPDH is used as the loading control. Two other experiments gave essentially the same results (data not shown).
(TIF)

Figure S4 **Both low glucose and amino acid restriction enhance PGC1α expression.** RNA was extracted from HEK cells maintained for 72 h in HG, LG, or Gal medium,+or – AA. PGC1α mRNA levels were quantified by Q-(RT)-PCR using GAPDH as a reference and normalized to HG+AA. N = 6 experiments, each in triplicate; data were analyzed using the student’s unpaired two-tailed *t*-test, * P<0.05, NS, not significant. Error bars are s.e.m.
(TIFF)

Figure S5 **RNAi against two proteins that contribute to mitochondrial translation induces an increase in the cytosolic 18S ribosomal RNA.** METTL17 and CHCHD1 co-purify with C4ORF14, which is tightly linked to the small subunit of the mitochondrial ribosome [44]. When either is repressed by RNA interference in human osteosarcoma cells, mitochondrial protein synthesis is severely compromised (He and Holt, manuscript in preparation). The total cellular RNA and proteins were extracted from human osteosarcoma cells treated with double-stranded RNAs targeting CHCHD1 and/or METTL17 or a scrambled RNA control. (**A**) Mitochondrial (12S and 16S) and cytosolic (18S) rRNA levels were determined by Q-(RT)-PCR with GAPDH as a reference, as previously described [39]. Error bars are one standard deviation from the mean, n = 3 experiments. Two-tailed unpaired student’s *t*-test (P<0.01 **, P<0.001 ***, not significant NS). **(B)** The abundances of the CHCHD1, METTL17, and GAPDH proteins were determined by immunoblotting. The Sigma Mission Pre-designed siRNAs (5′-3′) for METTL17 were (a) GUUCAAACCUUAUGGCGUATT (sense) and UACGCCAUAAGGUUUGAACTT (antisense); and (b) CAGUUAUUGCUA CUUGGGATT (sense) and UCCCAAGUAGCAAUAACUGTT (antisense). For CHCHD1, the siRNAs were (1) AACCUCUCAUUCUAGCUAATT (sense) and UUAGCUAGAAUGAGAGGUUTA (antisense); and (2) GGAGUUUACUUCCA AAUAATT (sense) and UUAUUUGGAAGUAAACUC CCA (antisense).
(TIFF)

Figure S6 **The replacement of glucose with galactose induces an increase in mtDNA copy number.** The chart shows the estimated mtDNA copy number; for HEK cells grown for 72 h in galactose (Gal) and amino acids, compared to the same cell type grown in high glucose (HG) with amino acids. [The data are derived from Fig. 4F]. n = 6; error bars represent the s.e.m. Transfer to galactose medium was associated with a significant increase in mtDNA copy number based on the unpaired student’s *t*-test, *** = p<0.001.
(TIFF)

Table S1 **The amino acid composition of DMEM defined as containing amino acids, none of which were present in DMEM minus amino acids.**
(TIFF)
