# Supplementary figures and images for: Amino Acid Starvation Has Opposite Effects on Mitochondrial and Cytosolic Protein Synthesis
Source: PLoS One. 2014 Apr 9;9(4):e93597. doi: 10.1371/journal.pone.0093597 (PMC3981720; doi:10.1371/journal.pone.0093597)

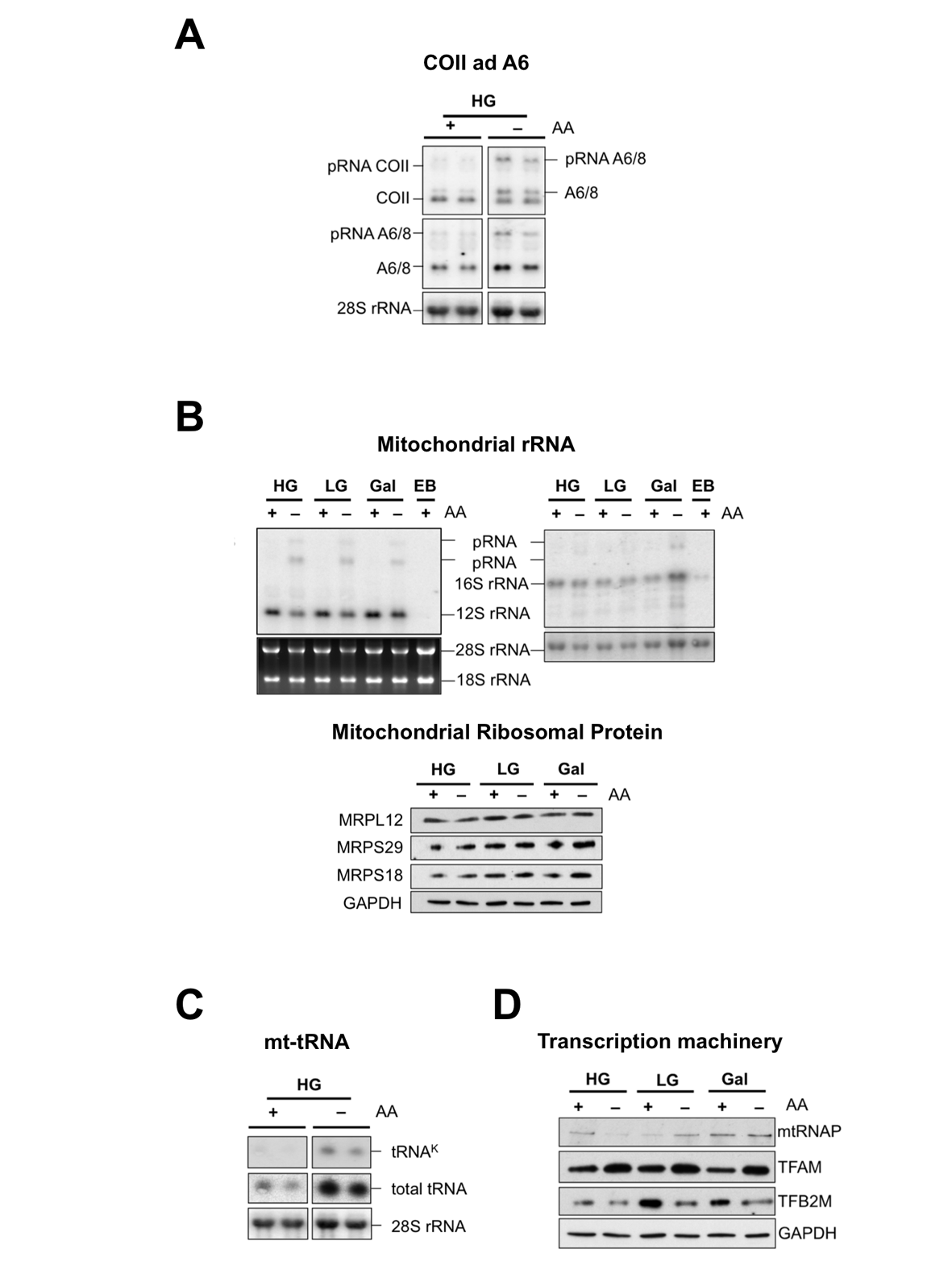

Supplement: Figure S3 — Effects of amino acid deprivation on mitochondrial RNAs and proteins involved in mitochondrial transcription. RNA and proteins were extracted from HEK cells. The RNA was fractionated by 1.2% agarose gel electrophoresis, transferred to nylon membranes, and hybridized with probes corresponding to mRNAs (A), rRNAs (B), or tRNAs (C). The proteins were immunoblotted using antibodies against (B) three mitochondrial ribosomal components (MRPs) or (D) the core transcription apparatus. (A) COII – cytochrome c oxidase subunit II mRNA, A6/A8– the single mature mRNA that encodes two subunits of ATP synthase. (B) 16S and 12S rRNAs are the RNA elements of mitochondrial ribosomes. (C) To gain an overall impression of the level of tRNAs in cells grown with or without amino acids, the portion of the membrane where tRNAs reside was hybridized to two labeled probes that together span the entire mitochondrial genome, as previously described [43]. In (A) and (C), the images for+and – amino acids derive from different portions of the same gel; additional samples (not shown) occupied the intervening lanes. (D) Immunoblotting for HEK cellular proteins indicated that amino acid deprivation had no effect on the steady state level of the mitochondrial RNA polymerase, in contrast to TFAM and TFB2M. GAPDH is used as the loading control. Two other experiments gave essentially the same results (data not shown). (TIF) [file pone.0093597.s003.tif]

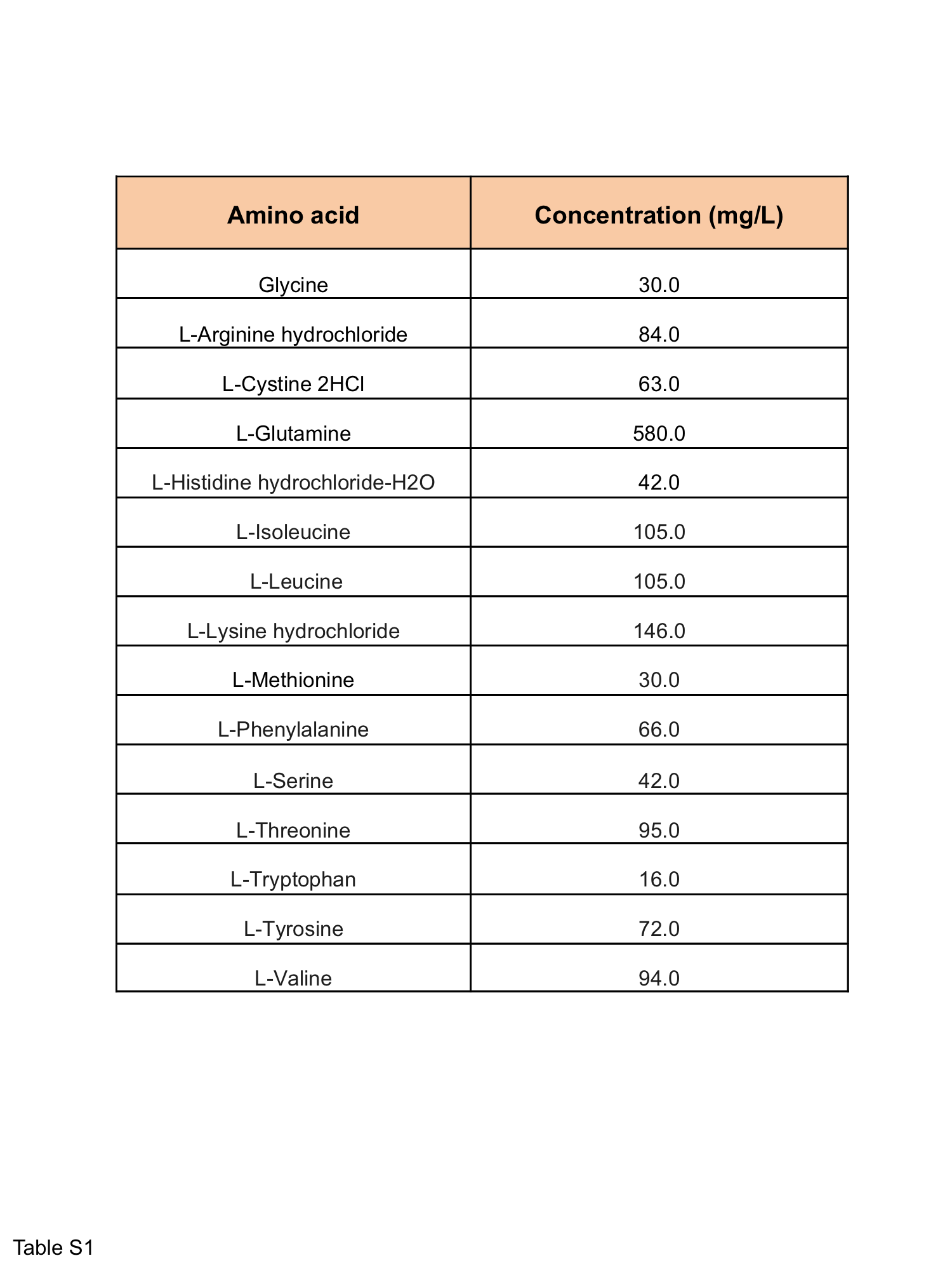

Supplement: Table S1 — The amino acid composition of DMEM defined as containing amino acids, none of which were present in DMEM minus amino acids. (TIFF) [file pone.0093597.s007.tiff]
